# Using Systematized Nomenclature of Medicine clinical term codes to assign histological findings for prostate biopsies in the Gauteng province, South Africa: Lessons learnt

**DOI:** 10.4102/ajlm.v9i1.909

**Published:** 2020-09-28

**Authors:** Naseem Cassim, Ahsan Ahmad, Reubina Wadee, Jaya A. George, Deborah K. Glencross

**Affiliations:** 1 National Health Laboratory Service(NHLS), National Priority Programme, Johannesburg, South Africa; 2 Department of Molecular Medicine and Haematology, Faculty of Health Sciences, University of the Witwatersrand, Johannesburg, South Africa; 3 Department of Urology, Faculty of Health Sciences, University of the Witwatersrand, Johannesburg, South Africa; 4 Department of Anatomical Pathology, Faculty of Health Sciences, University of the Witwatersrand, Johannesburg, South Africa; 5 Department of Chemical Pathology, Faculty of Health Sciences, University of the Witwatersrand, Johannesburg, South Africa

**Keywords:** prostate biopsy, Systemized Nomenclature of Medicine, SNOMED, morphology, topography, prostate cancer and adenocarcinoma

## Abstract

**Background:**

Prostate cancer (PCa) is a leading male neoplasm in South Africa.

**Objective:**

The aim of our study was to describe PCa using Systemized Nomenclature of Medicine (SNOMED) clinical terms codes, which have the potential to generate more timely data.

**Methods:**

The retrospective study design was used to analyse prostate biopsy data from our laboratories using SNOMED morphology (M) and topography (T) codes where the term ’prostate’ was captured in the narrative report. Using M code descriptions, the diagnosis, sub-diagnosis, sub-result and International Classification of Diseases for Oncology (ICD-O-3) codes were assigned using a lookup table. Topography code descriptions identified biopsies of prostatic origin. Lookup tables were prepared using Microsoft Excel and combined with the data extracts using Access. Contingency tables reported M and T codes, diagnosis and sub-diagnosis frequencies.

**Results:**

An M and T code was reported for 88% (*n* = 22 009) of biopsies. Of these, 20 551 (93.37%) were of prostatic origin. A benign diagnosis (ICD-O-3:8000/0) was reported for 10 441 biopsies (50.81%) and 45.26% had a malignant diagnosis (*n* = 9302). An adenocarcinoma (8140/3) sub-diagnosis was reported for 88.16% of malignant biopsies (*n* = 8201). An atypia diagnosis was reported for 760 biopsies (3.7%). Inflammation (39.03%) and hyperplasia (20.82%) were the predominant benign sub-diagnoses.

**Conclusion:**

Our study demonstrated the feasibility of generating PCa data using SNOMED codes from national laboratory data. This highlights the need for extending the results of our study to a national level to deliver timeous monitoring of PCa trends.

## Introduction

Prostate cancer (PCa) was a leading male cancer in South Africa in 2012, while globally it is the second most frequently diagnosed neoplasm.^1^ A 2012 global cancer study reported an estimated age-specific incidence rate of 67.9 per 100 000 for South Africa, with an associated mortality rate of 26.4 per 100 000.^1,2^ Presentation with highly aggressive PCa in African men in South Africa has been described.^3,4^

A national cancer control programme was recommended by the World Health Organization, with the aim of identifying priorities and assigning resources to sustain progress towards the reduction of cancer incidence and mortality as well as to improve the quality of life for patients with cancer.^5^ This would be attained through the equitable implementation of evidence-based strategies for prevention, early detection, treatment and palliative care, in the context of optimal use of limited healthcare resources.^6^ The 2013–2020 Global Action Plan for the Prevention and Control of Non-Communicable Diseases aims to achieve a 25% reduction in the relative risk of premature mortality from cancers and other non-communicable diseases.^7^ This initiative emphasises the importance of both surveillance and disease registries that should be integrated into existing health information systems to improve the availability of high-quality data.^7^ Current cancer registry reporting is not integrated into any hospital information system. The purpose of a cancer registry is to establish and maintain a cancer incidence reporting system that informs planning of cancer control programs.^8^ Cancer registries should typically publish annual data within 28 months after the close of the year in which the incident case was diagnosed.^9^ A delay in cancer registry reporting is a major limitation for understanding PCa trends.^9^

The National Cancer Registry of South Africa is a passively reported registry that used the International Classification of Diseases for Oncology (ICD-O-3) — recommended international methodology — to manually code pathology reports.^10,11,12^ The ICD-O-3 describes both the anatomical site and cell type and behaviour (malignant or benign biopsy).^13^ The National Cancer Registry (NCR) had only reported data for 2014 in 2018.^14^ Cases are manually coded, and this results in a reporting delay. Based on the surveillance, Epidemiology, and End Results programme standard, the 2015 report should have been published by 2018.^9^ The programme itself, on which the South African reporting standard is based, is comprised of 11 registries in five states and six metropolitan areas in the United States which generate annual cancer data approximately 28 months after diagnosis.^8^

Both internationally and nationally, cancer surveillance is defined as an ongoing, timely, and systematic collection and analysis of cancer data to assess risk factors, screening, diagnosis, and cancer incidences and deaths.^9^ The aim of surveillance is to analyse and disseminate cancer data to identify challenges and opportunities in the delivery of timeous cancer control programmes.^9^

The cancer registry reporting is a time-intensive process requiring trained coders to review each narrative biopsy report individually and to manually add the applicable ICD-O-3 codes to the reporting system.^15^ This can take hundreds of hours of manual database building to report PCa data. The coders would have to add both topography and morphology ICD-O-3 codes for each narrative biopsy report reviewed.^15^ This manually intensive process prolongs time between diagnosis and reporting of identified PCa cases for surveillance. PCa reporting is required at least 28 months after diagnosis (~2 years) to understand changes in incidence.^8^ Without timely data this would not be possible. Therefore, new approaches are required to reduce or automate the coding process to provide more timely cancer data.

Some studies have used approaches such as natural language processing and text mining.^16,17,18^ For our study, we decided to use the Systematized Nomenclature of Medicine (SNOMED) clinical terms.^19^ The Systematized Nomenclature of Medicine is a comprehensive and precise health terminology used globally that incorporates a structured list of health terms or concepts.^19,20^ One of the benefits of using SNOMED is that codes can be mapped to other coding systems to facilitate interoperability.^19^ All references to SNOMED relate to SNOMED CT.^21^ In an Anatomical Pathology setting, SNOMED CT is used to capture the histological finding in the form of morphology (M) and topography (T) codes. The M and T codes are captured directly in the laboratory information system (LIS) by the pathologist after examining prostate cores. The same histological findings are also reported as a narrative pathology report.^19^ The M and T code(s) are captured separately in defined test items in the LIS, which has a dictionary of all the SNOMED codes that may be reported and the anatomical pathologist selects the appropriate codes to add based on the histological findings. For each biopsy, more than one SNOMED M/T code may be captured. For example (personal communication, Vreede H, August 12, 2017, sharing the laboratory information system SNOMED CT code table extract), for a biopsy of prostatic origin with an adenocarcinoma finding, the following codes would be reported (description in brackets); (1) T-28 000 or T-92 000 (Prostatic structure), (2) M-80 003 (Malignant neoplasm, primary) and (3) M-81 403 (Adenocarcinoma, no subtype).

Studies outside South Africa have used SNOMED codes to transform laboratory and other reported data for cancer registry reporting.^22^ A good example is the Danish PCa registry that analysed SNOMED data for biopsies with PCa, that is histologically verified.^22^ This study confirmed that the SNOMED codes generated clinically useful data.^22^

Our study described here is the first attempt in South Africa to investigate reporting PCa using SNOMED codes by collating this information contained in the national laboratory data repository. It is anticipated that this could, in future strengthen surveillance activities. Additionally, using the SNOMED codes to report on patients without PCa could provide important presentation information that is currently poorly understood.

The majority of local studies have manually coded biopsy reports to extract PCa information. The development of SNOMED CT lookup tables have the potential to automate this process and improve timely PCa reporting. The objective of this study was to describe the methodology used to report PCa data using SNOMED CT lookup tables.

## Methods

### Ethical considerations

Ethics clearance for this study was obtained from the University of the Witwatersrand (M170419). This study used national laboratory data that does not contain any patient identifiers. No patient recruitment was required.

### Study design

This was a retrospective descriptive study that analysed prostate biopsy data between 2006 and 2016 for men ≥ 30 years in the Gauteng province.

### Data extraction and preparation

Retrospective prostate biopsy data for the period 2006–2016 were extracted from the national health laboratory repository of patient-related data where the term ‘prostate’ was captured in the narrative pathology report. Simple text mining approaches were used in the Netezza Aginity (Marlborough, Massachusetts, United States) query tool which employed pattern matching by fuzzy string search function.^23^ Two data extracts were received: (1) prostate biopsy, and (2) chained M and T code(s) captured for each biopsy.

The prostate biopsy extract included the following variables: (1) episode number, (2) unique patient identifier (generated using a probabilistic matching algorithm^24^), (3) age, (4) gender, (5) race (where populated), (6) facility code, (7) facility name, (8) reviewed date, and (9) biopsy results text (unstructured narrative report detailing histological findings).

The SNOMED data extract included the following variables: (1) episode number, (2) chained (comma separated) M code(s) (comma delimited), e.g. M-00 100, M-43 000, M-72 450, and (3) chained T code/s, e.g. T-92 000, T-74 000).

**FIGURE 1:**
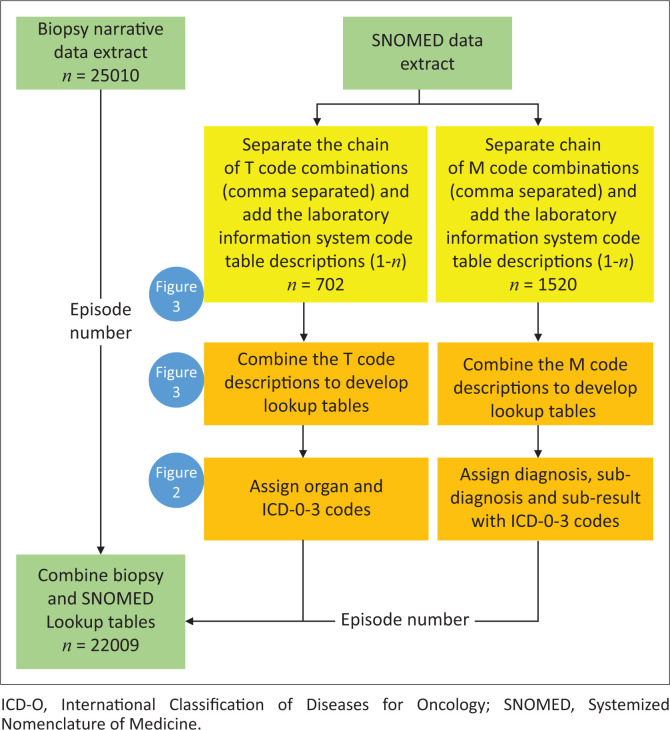
High-level overview of the steps taken to code the unique chained Systemized Nomenclature of Medicine (SNOMED) code combinations extracted from the biopsy narrative data extract. There were two separate SNOMED data extracts from the laboratory information system: morphology (M) and topography (T). The colour coding indicates the various processes; (1) green: data extracts, (2) yellow SNOMED code manipulation in preparation for lookup table development, and (3) orange: lookup tables with coded variables. The extracted unique chained SNOMED code combinations were used to prepare the two lookup tables to generate the following new coded variables; (1) organ, (2) organ ICD-O-3, (3) diagnosis, (4) diagnosis ICD-O-3, (5) sub-diagnosis, (6) sub-diagnosis ICD-O-3, and (7) sub-result (for an inflammation sub-diagnosis). The number of biopsies is indicated for each data extract. The blue circles indicate which figures provide additional details on each step.

**FIGURE 2:**
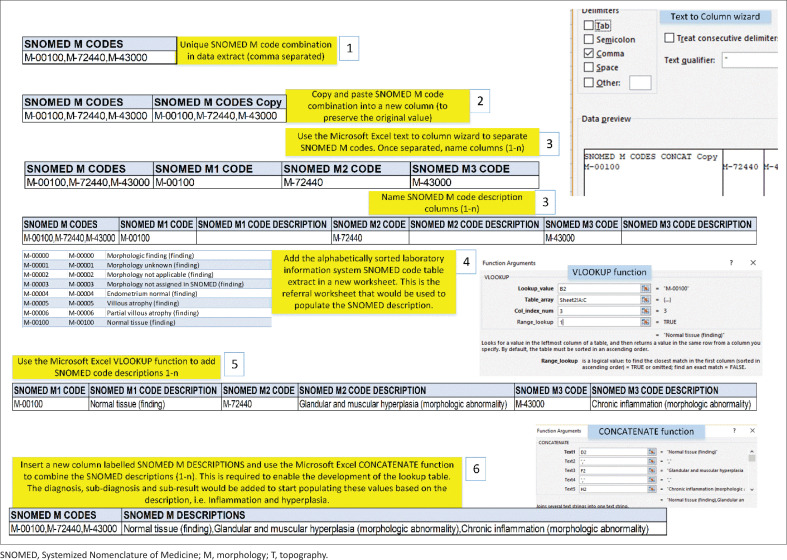
Six-step procedure used to transform the chained comma separated Systemized Nomenclature of Medicine M codes into individual columns (leaving the original value intact) to add the laboratory information system code table descriptions (in preparation for lookup table development). The same procedure was conducted for T codes. The manipulation was achieved using standard Microsoft Excel functions (screenshots included next to each step). The steps are as follows: (1) copy unique chained codes to a new worksheet and then copy and paste to a new column for processing (leaving the original values intact), (2) use the Microsoft Excel text to column function to separate the chained codes and name new columns, e.g. M Code 1-n, (3) insert a new column next to each code column and label as a description column, e.g. M Code Descr 1-n, (4) add the alphabetically sorted laboratory information system systemized nomenclature of medicine code description in a new worksheet, (5) use the Microsoft Excel VLOOKUP function to add the code description (range lookup set at 1 for an exact match), e.g. M-00 100 code description is ‘Normal tissue (finding)’, and (6) Microsoft Excel CONCATENATE function used to combine code descriptions combined in a new column.

**FIGURE 3:**
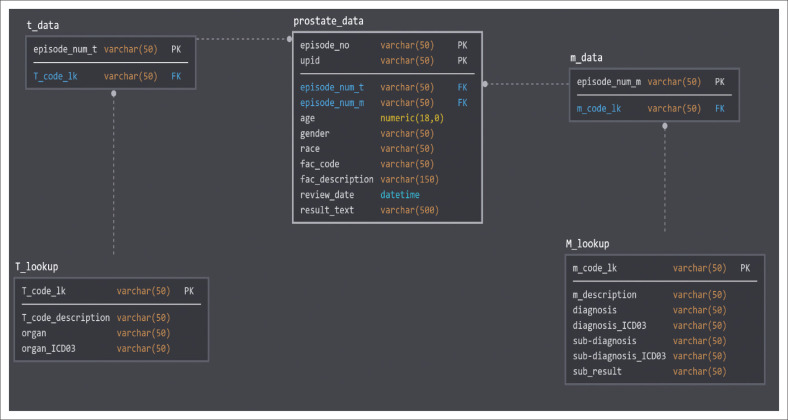
Relational database diagram describes how the various tables were joined (left outer). For each table, the primary and foreign keys are provided. The lookup tables contain only the unique Systemized Nomenclature of Medicine code combinations. The lines indicate a relationship join between tables. Once the table joins are implemented, variables from any table can be reported. Structured query language could be used to combine the required data for analysis.

From the prostate biopsy data extract, the unique SNOMED CT code combinations were extracted, and a lookup table was developed (Figure 1). The lookup tables were developed in a two-step process: (1) code manipulation to combine descriptions, and (2) coding the lookup tables. The blue circles in the figure indicate which figures provide additional details for each step, that is: data manipulation (Figure 2) and query to combine data extracts and lookup tables in a single database query (Figure 3).

### Combining Systemized Nomenclature of Medicine descriptions for the lookup table

The chained SNOMED codes were provided in a format that could not readily be analysed and had to be separated into individual columns with the applicable descriptions added from the LIS code table. Data were prepared using Microsoft Excel (Microsoft Corporation, Redmond, Washington, United States)^25^ (Figure 2). The unique code combinations and descriptions were extracted for the development of the lookup table.

### Coding the Systemized Nomenclature of Medicine M and T lookup tables

We used the prepared M and T code descriptions to start populating the lookup tables. For the M lookup table, we used each unique code description combination to populate the following new variables: (1) diagnosis, (2) diagnosis ICD-O-3 code, (3) sub-diagnosis, (4) sub-diagnosis ICD-O-3 code, and (5) sub-result with guidance from an anatomical pathologist and a urologist. The team reviewed each code combination and assigned values to be captured in the lookup table. An example of the coding is provided for four biopsies in Table 1. We captured the matching ICD-O-3 codes for predominantly malignant findings. The diagnosis reports the overall biopsy finding, whereas the sub-diagnosis was used to differentiate the diagnosis, e.g. Benign, negative for malignancy (ICD-O-3: 8000/0) and ‘Hyperplasia’, respectively (Episode A). Assigning the malignant code descriptions was fairly easy, but we struggled with benign findings given the number of findings reported and the order thereof. To clarify coding, we defined the reporting order to assign a benign sub-diagnosis as follows: (1) inflammation, (2) hypertrophy, (3) hyperplasia, (4) edema, (5) atrophy, and (6) adenosis. This made it easier to assign these finding in logical order. The sub-result was used to identify the type of inflammation reported, e.g. acute, chronic, granulomatous, etc. Similarly, the T code lookup table reported the organ (Prostate/Other) and associated ICD-O-3 code (C61.9 for Prostate).

**TABLE 1:** Example of four prostate biopsies where the systemized nomenclature of medicine code descriptions were assigned a diagnosis and sub-diagnosis including the allocation of ICD-O-3 codes.

Episode no.	SNOMED M codes (T Code)	Biopsy result text	Date of review	Gleason score	SNOMED M descriptions	Diagnosis (ICD-O-3)	Sub-diagnosis (ICD-O-3)
A	M-72 450,M-00 100 (T-92 000)	†	2009/10/19	N/A	Adenofibromyomatous hyperplasia (morphologic abnormality) Normal tissue (finding)	Benign/negative for malignancy (8000/0)	Hyperplasia
B	M-72 000 (T-92 000)	‡	2013/03/12	N/A	Hyperplasia (morphologic abnormality)	Benign/negative for malignancy (8000/0)	Hyperplasia
C	M-00 100,M-81 403 (T-92 000)	§	2014/02/16	4 + 3 = 7	Normal tissue (finding) Adenocarcinoma, no subtype (morphologic abnormality)	Neoplasm, malignant (8000/3)	Adenocarcinoma (8140/2)
D	M-71 000,M-40 000 (T-92 000)	¶	2016/10/28	N/A	Hypertrophy (morphologic abnormality) Inflammation (morphologic abnormality)	Benign/negative for malignancy (8000/0)	Inflammation and hypertrophy

Note: The biopsy result text is also provided. Episode numbers are anonymised.

SNOMED, Systemized Nomenclature of Medicine; M, morphology; T, topography; ICD-O, v International Classification of Diseases for Oncology; N/A, not applicable.

† , episode number: a clinical history: an 86-year-old male patient with a prostate specific antigen = 14.1. Microscopy: sections of the specimen confirm the presence of multiple cores of prostatic tissue with features of benign fibromuscular and adenomatous hyperplasia. Diagnosis: prostate – benign fibromuscular and adenomatous hyperplasia.

‡ , episode number: b clinical history: a 57-year-old man. Re-turp. Macroscopy: 8.5 g of chips received, all processed. Microscopy: sections show fragments of prostate tissue exhibiting features of benign nodular glandular and stromal hyperplasia and mild chronic prostatitis. There is no evidence of high-grade prostatic intraepithelial neoplasia or infiltrating malignancy. Pathological diagnosis: Turp: – prostatic hyperplasia and chronic prostatitis – no evidence of malignancy.

§ , episode number: c clinical history: an 82-year-old patient with prostate specific antigen = 273. Hard, multinodular prostate. Trus biopsy done. macroscopy: twelve biopsies, the longest 1.8 cm. Microscopy: histological examination shows numerous cores of prostatic tissue, most of which contain extensive infiltration by adenocarcinoma, Gleason 4, 3. Focally is perineural invasion seen. Pathological diagnosis: prostate gland: extensive involvement by adenocarcinoma, Gleason 4, 3 focally perineural invasion is seen.

¶ , episode number: d clinical details: the patient is a 61-year-old male. The patient has a prostate specific antigen level of 10.4. Macroscopy: 13 cores, the longest of which measured 11 mm and the shortest 2 mm. pathological diagnosis: prostate: (1) sections show representation predominantly of seminal vesicles. (2) There is focal representation of rectal mucosa. (3) Foci of chronic inflammation are identified. (4) Features of benign prostatic enlargement (BPE) are present. There is no evidence of prostatic intraepithelial neoplasia (pin) or invasive adenocarcinoma in the sections examined.

### Combining the lookup tables with the prostate biopsy data

Microsoft Access (Microsoft Corporation, Redmond, Washington, United States) was used to combine the datasets in a single query: (1) prostate data extract table, (2) SNOMED M lookup table, and (3) SNOMED T lookup table. Tables were combined using a left outer join in a relational database (Figure 2).^26^ This join type ensures that all rows from the prostate data extract table were populated with only the matching values from the other tables reported using referential integrity (Table 1).^26,27^ This query contained all the variables for the data analysis.

### Systemized Nomenclature of Medicine M code descriptive analysis

The number of prostate biopsies with an M and T code populated was assessed as a contingency table using SAS Enterprise Guide 7.1 (SAS Institute Incorporated, Cary, North Carolina, United States).^28^ Descriptive analysis was conducted for prostatic biopsies with the M code captured. Test volumes for the top 10 M code combinations with their chained descriptions were reported (in descending order). The number of biopsies of prostatic origin was also reported.

### Descriptive analysis of diagnosis and sub-diagnosis

The diagnosis and sub-diagnosis volumes were reported for biopsies with M code populated of prostatic origin. For each diagnosis, the sub-diagnoses were then reported. Where more than 10 sub-diagnoses were reported, the first 10 were reported and the remaining grouped as ‘Other’.

## Results

Using our methodology, 25 010 biopsy results were extracted and analysed. For the lookup tables, there were unique 1520 M and 702 T code combinations.

### Systemized Nomenclature of Medicine M and T code descriptive analysis

M codes were provided for 22 195/25 010 biopsies (89%; Table 2). The T code was provided for 24 546/25 010 (98%) biopsies. There were 22 009/25 010 biopsies with both an M and T code populated (88%). There were 2815/25 010 (11%) biopsy reports that could not be analysed using lookup tables; they did not have both M and T codes (Table 2). Descriptive analyses were conducted for 20 551/25 010 (82%) biopsies of prostatic origin. Of the 22 195 biopsies with M codes, M-40 000 (‘Inflammation’) was the most commonly reported sub-diagnosis (*n* = 3400; 15.3%). This was followed by two adenocarcinoma combinations: M-81 403 (*n* = 2677; 12.1%) and M-81 403, M-80 003 (*n* = 2166; 9.8%) (Table 3).

**TABLE 2:** Contingency table to assess the percentage of Systemized Nomenclature of Medicine M and T codes populated using the mapping table for prostate biopsies between 2006 and 2016 in the Gauteng province, South Africa.

Status	T Code(s) captured		T Code(s) not captured		Total	
	*n*	%	*n*	%	*n*	%
M Code/s captured	22 009	88	186	1	22 195	89
M Code/s not captured	2537	10	278	1	2815	11
**Total**	**24 546**	**98**	**464**	**2**	**25 010**	**100**

M, morphology; T, topography.

**TABLE 3:** Top 10 most commonly requested Systemized Nomenclature of Medicine M code combinations from the prostate biopsy data between 2006 and 2016 in the Gauteng province, South Africa.

M codes	M code descriptions	Total	
		*n*	%
M-40 000	Inflammation (morphologic abnormality)	3440	15.5
M-81 403	Adenocarcinoma, no subtype (morphologic abnormality)	2677	12.1
M-81 403, M-80 003	Adenocarcinoma, no subtype (morphologic abnormality) Malignant neoplasm, primary (morphologic abnormality)	2166	9.8
M-00 100	Normal tissue (finding)	1668	7.5
M-80 003, M-81 403	Malignant neoplasm, primary (morphologic abnormality) Adenocarcinoma, no subtype (morphologic abnormality)	1596	7.2
M-00 100, M-81 403	Malignant neoplasm, primary (morphologic abnormality) Adenocarcinoma, no subtype (morphologic abnormality)	1299	5.9
M-00 100, M-72 440	Normal tissue (finding) Glandular and muscular Hyperplasia (morphologic abnormality)	1081	4.9
M-80 003	Malignant neoplasm, primary (morphologic abnormality)	706	3.2
M-09 350	Morphologic description only (finding)	394	1.8
M-72 440	Glandular and muscular hyperplasia (morphologic abnormality)	370	1.7

M, morphology.

### Descriptive analysis of diagnosis and sub-diagnosis

Using this approach, we noted 10 441 (50.81%) biopsies with a benign diagnosis (ICD-O-3:8000/0) and 9302 (45.26%) biopsies with a malignant diagnosis (8000/3). Atypia was reported for 760 biopsies (3.7%). An uncertain (whether benign or malignant) diagnosis (8000/1) was reported for 48 (0.23%) biopsies (Table 4).

**TABLE 4:** Descriptive analysis of diagnosis and sub-diagnosis where both a Systemized Nomenclature of Medicine M and T code are populated of prostatic origin between 2006 and 2016 in the Gauteng province, South Africa.

Lookup table value	*n*	%
**Diagnosis**		
Benign/negative for malignancy (8000/0)	10 441	50.81
Neoplasm, malignant (8000/3)	9302	45.26
Atypia/dysplasia (8000/0)	760	3.70
Neoplasm, uncertain whether benign or malignant (8000/1)	48	0.23
Total	20 551	-
**Sub-diagnosis**		
**Benign/negative for malignancy**		
Inflammation	4075	39.03
No pathologic diagnosis	2809	26.90
Hyperplasia	2174	20.82
Inflammation and hyperplasia	788	7.55
Inflammation and hypertrophy	123	1.18
Atrophy	120	1.15
Hypertrophy	82	0.879
Inflammation and atrophy	63	0.60
Hyperplasia and atrophy	55	0.53
Inflammation, hypertrophy and hyperplasia	31	0.30
Other	121	1.16
Total	10 441	-
**Neoplasm, malignant sub-diagnosis**		
Adenocarcinoma (8140/3)	8201	88.16
Carcinoma (8010/3)	408	4.39
Malignant neoplasm (8000/3)	693	7.45
Total	9302	-
**Atypia/dysplasia sub-diagnosis**		
Atypia	616	81.05
Dysplasia	87	11.45
Atypia/dysplasia	44	5.79
High grade intraepithelial lesion (8148/2)	13	1.71
Total	760	-
**Neoplasm, uncertain whether benign or malignant sub-diagnosis**		
Neoplasm, uncertain whether benign or malignant (8000/1)	48	0.23

Note: Where appropriate, the ICD-O-3 codes are provided in brackets.

M, morphology; T, topography; ICD-O, International Classification of Diseases for Oncology.

Inflammation was reported as a sub-diagnosis for 4075 benign biopsies (39.03%). This was followed by no pathologic diagnosis and hyperplasia at 26.90% (*n* = 2809) and 20.82% (*n* = 2174) respectively. Inflammation and hyperplasia were reported in two combinations contributing 7.85% of the top 10 benign sub-diagnoses. Cumulatively, the top 10 sub-diagnoses reported represented 98.8% (*n* = 10 320) (Table 4) of all benign diagnoses. The majority of samples with a malignant diagnosis reported an adenocarcinoma (8201, 88.16%) sub-diagnosis. There were 408 biopsies with a carcinoma (4.39%) sub-diagnosis. The malignant neoplasm sub-diagnosis was reported for 693 (7.45%) biopsies where it was not possible to differentiate the tissue type, reporting predominantly the M-80 003 code (Malignant neoplasm, primary) (personal communication, Vreede H, August 12, 2017). The majority of biopsies with an atypia/dysplasia diagnosis reported an atypia sub-diagnosis (*n* = 616; 81.05%) followed by dysplasia (*n* = 87; 11.45%). Only 1.71% of biopsies with an atypia/dysplasia diagnosis reported a high grade intraepithelial lesion (*n* = 13). Only 48 (0.23%) biopsies reported an uncertain sub-diagnosis (8000/1).

## Discussion

We showed that it was possible to automate prostate biopsy reporting using a commonly available relational database (Microsoft Access) and SNOMED lookup tables in the Gauteng province. The use of ICD-O-3 codes for malignant findings facilitate PCa reporting similar to cancer registries.^13^

To routinely automate the registration and surveillance of PCa in South Africa, the lookup tables developed for this study would need to be introduced to the corporate data warehouse. Lookup tables are routinely used by the Corporate Data Warehouse (CDW) to transform laboratory data for reporting, for example the HIV serology results reported as ‘NEG’, ‘N’ or ‘NEGATIVE’ are transformed to a single value (‘NEG’) for uniform reporting.^28^ The benefit of this mechanism for PCa reporting is that as biopsies are reported in the LIS, the data replicated to the CDW will be conformed to report the biopsy diagnosis and sub-diagnosis within three months of diagnosis.

Lookup tables would facilitate a constant feed of analysed PCa data to the South African NCR to ensure timeous reporting. Over time, any new SNOMED code combinations identified would have to be added to the lookup table. By providing this data at shorter intervals, it would be possible to triangulate against other local data sources (NCR and other). Triangulation is the process used in public health to review and interpret data from multiple sources that answer the same question for decision making.^30^ Unpublished data from this study revealed that between 2012 and 2016, PCa incidence has increased from 44.92 to 57.31 per 100 000 compared to 46.53 reported by the NCR in 2012.^31^

Another advantage of this approach is that PCa data from other African countries using a LIS could also be analysed using the developed lookup tables to dramatically improve PCa reporting across Africa. Antoni et al. assessed the methods used for reporting the 2018 Global Cancer Statistics estimates.^32^ For 14/51 African countries, PCa incidence estimates were based on simple average rates from neighbouring countries (27%).^32^ For South Africa, projections of national incidence were sourced from the NCR.^32^ The SNOMED lookup tables have the potential to improve both national and regional PCa incidence reporting across the African continent providing more accurate data. With better data, cancer control initiatives could be better mobilised.

The principles applied in our study could also be implemented for other cancers. The SNOMED codes are captured for all cancers of public health importance routinely. Similar lookup tables could be developed to report on lung, breast and cervical cancers with incidence rates of 17.3, 49.0 and 13.5 per 100, 1000 respectively in 2018 in South Africa.^33^

The approach described in our study is not unique. Similar approaches have been employed in the Danish cancer registry, where data reported for 161 525 biopsies for the period 1995–2011 were undertaken using SNOMED codes.^22^ The Danish cancer registry predominantly reported data for PCa, whereas our study reported data for negative biopsies as well. The combination of the lookup tables reported in our study with text mining to extract the Gleason score reported in an unpublished study could be used to report data similar to the Danish cancer registry, for example diagnosis of ‘Neoplasm, malignant’, sub-diagnosis of ‘Adenocarcinoma’ and a Gleason score of 3 + 3 = 6 would be coded as ‘bGS3+3’.^22^

It is important to provide up to date PCa data at both the national, provincial, district and health facilities levels to identify hotspots where programmatic interventions are required. SNOMED lookup tables could be used to focus programmatic interventions for geographic areas with a higher PCa burden. Similar initiatives using laboratory data have been undertaken locally for HIV and tuberculosis services.^24,34,35^ An example is the World Bank report that described spatial clustering analysis of HIV viral load suppression at the national, provincial, district, sub-district and health facility levels.^24^ This study indicated that national laboratory data stored in the CDW has the potential to provide important strategic information on the quality and reach of the antiretroviral therapy programme by highlighting the geographical variation in the proportion of patients virally suppressed.^24^ This information can be accessed by healthcare workers using the epidemiological dashboard developed to identify health facilities that are performing poorly.^24,34^ Similar work has also been published using cluster of differentiation 4 data to highlight areas where HIV-positive patients presenting for care have a higher burden of advanced disease.^34^ The assimilation of health data into workable and user-friendly dashboards has had a big impact on how health data is used locally.^36,37,38^ In the medium term, it would be possible to develop a PCa epidemiological dashboard similar to the HIV example mentioned to report PCa trends by age, race group and geographical boundaries routinely. The PCa dashboard could facilitate the reporting of the number of incident cases. The dashboard could also provide insights into how and where health care services are being accessed to inform both guideline changes and programmatic improvements to facilitate equitable access to care across the country. It could also provide loss to follow up and waiting time data for patients who had presented with an elevated prostate specific antigen and who were confirmed with PCa histologically using the CDW probabilistic matching algorithm.^29^

Data reported in our study additionally demonstrated functionality by providing data for benign histological findings, potentially useful to identify trends for patients diagnosed with chronic inflammation who eventually progress to PCa. While the ICD-O-3 codes are particularly important for PCa cases, the importance of additional benign findings is especially important for inflammation and hypertrophy that have been shown to be linked with many other cancers.^39,40^ Several studies have indicated that chronic inflammation has a potential role in prostatic carcinogenesis and tumour progression.^39,41,42^ Nelson et al. reported that chronic or recurrent inflammation may play a role in the development of PCa.^43^ Using the data generated using the lookup tables, patients with chronic inflammation could be followed up to identify whether they progress to PCa in an African setting.

Finally, to improve SNOMED reporting, it is recommended that the M and T codes be defined as mandatory fields. This will ensure that 100% of biopsies include these codes. This will address the 12% of biopsies without this information. Future research includes extending the lessons learnt with PCa in the use of lookup tables to other common cancers, as patient-level data is already available in the CDW and could be easily unlocked for national cancer reporting.

### Limitations

One of the limitations is that the data for our study was limited to biopsies sent to the NHLS and did not include data from private sector laboratories and thus limits the generalisability of our study. As private sector laboratories also use the SNOMED code, discussions will be initiated with them to share PCa data for national reporting.

An additional limitation of our data is the 10% of biopsies excluded a SNOMED M or T code. Unfortunately, these fields are not mandatory and can be uncaptured. By amending rules and making these fields mandatory, all biopsies would prospectively be reviewed with at least one T and M code captured. The excluded data would also affect the generalisability of our study. We are not able to determine the findings for these biopsies. To address this gap, text mining and machine learning approaches are being investigated. A sample of the biopsy data will be used to train the machine learning models i.e. malignancy (1) and benign (0). The big data tools will be validated against well populated SNOMED data entailing grid search, *k*-fold cross validation, precision, recall and *F*-score. The combination of SNOMED, text mining and machine learning will hopefully address the missing data. With minor changes to the laboratory information system, this has the potential to report PCa histological findings for all biopsies.

### Key messages

Existing national laboratory data has been used for the first time in South Africa to report PCa diagnosis across a province using SNOMED lookup tables. This could be implemented across South Africa to provide timely PCa trends.

### Conclusion

Our study has demonstrated that it is possible to automate PCa reporting using SNOMED codes for 88% of biopsies. The value of national laboratory data as shown in our study can easily be extended to deliver the timely monitoring of PCa trends across South Africa and other African countries. This could also be applied to report data for other cancers of public health interest.
